# Identification of recurring protein structure microenvironments and discovery of novel functional sites around CYS residues

**DOI:** 10.1186/1472-6807-10-4

**Published:** 2010-02-02

**Authors:** Shirley Wu, Tianyun Liu, Russ B Altman

**Affiliations:** 1 23andMe, 1390 Shorebird Way, Mountain View, CA, USA; 2 Program in Biomedical Informatics, Stanford University, Palo Alto, CA, USA; 3 Department of Genetics, Stanford University, Palo Alto, CA, USA; 4 Department of Bioengineering, Stanford University, Palo Alto, CA, USA

## Abstract

**Background:**

The emergence of structural genomics presents significant challenges in the annotation of biologically uncharacterized proteins. Unfortunately, our ability to analyze these proteins is restricted by the limited catalog of known molecular functions and their associated 3D motifs.

**Results:**

In order to identify novel 3D motifs that may be associated with molecular functions, we employ an unsupervised, two-phase clustering approach that combines k-means and hierarchical clustering with knowledge-informed cluster selection and annotation methods. We applied the approach to approximately 20,000 cysteine-based protein microenvironments (3D regions 7.5 Å in radius) and identified 70 interesting clusters, some of which represent known motifs (*e.g*. metal binding and phosphatase activity), and some of which are novel, including several zinc binding sites. Detailed annotation results are available online for all 70 clusters at http://feature.stanford.edu/clustering/cys.

**Conclusions:**

The use of microenvironments instead of backbone geometric criteria enables flexible exploration of protein function space, and detection of recurring motifs that are discontinuous in sequence and diverse in structure. Clustering microenvironments may thus help to functionally characterize novel proteins and better understand the protein structure-function relationship.

## Background

Protein function and structure are inherently linked, with molecular interactions determined by the shape and energetics of the participating structures. Knowledge of structure is especially important for elucidating detailed molecular mechanisms of function for the development of disease therapeutics and pharmaceuticals. Galvanized by the Protein Structure Initiative, the field of structural genomics has begun to solve the structures of proteins in high-throughput [1-3]. By solving representative structures throughout protein structure space, researchers can more fully determine the relationship between protein structure and function [4]. Many of the solved structural genomics targets, however, lack annotation regarding the proteins' biological functions.

Numerous methods exist for predicting protein function computationally, with most using some kind of sequence or structure-based similarity to match the query protein to other proteins or trained models of known function. The most popular sequence-based methods employ Hidden Markov Models to detect matches to functional domains, such as Pfam [5] and SUPERFAMILY [6], or use regular expression patterns and shorter motifs, such as PROSITE [7] and PRINTS [8]. Structure-based methods include those that use structural alignments of the protein backbone (*e.g*. Dali [9]) or secondary structure elements (*e.g*. SSM [10]), those that match spatial residue geometry such as 3D templates [11], and those that use a combination of structural features more abstractly - for example, FEATURE [12].

Since most of these methods use prior knowledge in a supervised fashion, they are good at detecting functions that are already well characterized in proteins that bear similarity in sequence and/or structure to known proteins. By design, many unannotated structural genomics proteins have sequences with no detectable homology to known structures; these structures are, not surprisingly, often dissimilar in structure as well [13]. Consequently, most methods that rely on conserved similarities in sequence or structure may not successfully predict function for these proteins. Because structure is more conserved than sequence [14], structure-based methods may have more success [15,16], but even structure-based methods will struggle if structural similarity to known proteins is very low. In these cases, we have shown that an abstract 3D representation, called a microenvironment, can better model and predict functional sites [16,17].

Most methods for function annotation require the sequence or structure motif to be present in a continuous stretch of polypeptide. This requirement makes the discovery of convergent or highly divergent motifs more difficult. 3D templates - specifically the "reverse template" technique - can be used to describe recurring residue triads that are discontinuous in sequence, but this method has not been applied in a comprehensive manner across a large set of structures. Similarly, cleft- and patch-finding algorithms such as CASTp [18], PocketPicker [19], and PatchFinder [20] can identify interesting regions of protein structures, but have not been applied comprehensively to discover recurring motifs.

Unsupervised approaches are useful for discovering patterns and groups in data without prior information or training. A recent study by Manikandan and colleagues [21] clustered structural fragments from a fold-unique subset of the Protein Data Bank (PDB) [22] based on backbone angles, resulting in groups of fragments with similar conformations. They then associated each fragment with Gene Ontology (GO) terms [23] to produce significantly enriched functional labels for groups. Their findings support the idea that clustering of sub-structures can identify novel functional motifs.

In this work, we present an unsupervised procedure for clustering microenvironments in protein structures. The microenvironments are described using physicochemical properties radially averaged around a site of interest and therefore do not constrain residue identities or continuity in sequence or structure. We have used this representation previously to build robust, supervised models of protein function [12,16,24] and to perform a preliminary study of unsupervised clustering [25]. We have improved the clustering procedure by better defining the biological context and decreasing feature redundancy, and applied a discriminating cluster selection method to produce more coherent groups, which we then annotate using external knowledge from several sources.

We demonstrate that this approach, applied to a set of cysteine (CYS) residues from a subset of the PDB, is able to rediscover known functions, distinguish between functional sub-classes, make compelling functional site predictions for individual proteins, and identify novel groups of interesting microenvironments. We therefore show the value of representing protein structure and functional sites using microenvironment similarity. Cysteine is an interesting initial residue on which to focus because it plays diverse and widespread roles in biology, including in proteolysis, redox-catalysis, structural stability, and metal-binding [26].

## Results

**Figure 1:**
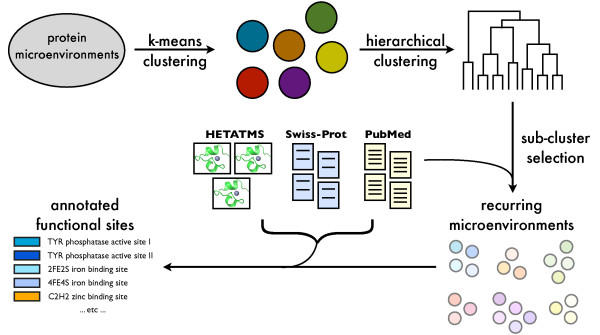
**Overview of functional site discovery approach**. Starting from thousands of protein microenvironments, we use k-means clustering to group them into coarse clusters. Each coarse cluster is then hierarchically clustered, and optimal clusters are identified using a scoring function that incorporates knowledge from scientific literature. These clusters are annotated using information from literature, Swiss-Prot records, and PDB HETATM data to produce novel individual site annotations and potentially novel functional motifs.

We used *k*-means followed by hierarchical clustering to group a set of cysteine-based microenvironments into clusters. To approximate the biological signal present in a cluster, we used a literature-based metric called functional coherence [27,28] which measures the degree to which a set of proteins shares similar literature. The resulting clusters were associated with descriptive terms derived from curated databases and literature abstracts associated with the proteins in each cluster. The overall approach is outlined in Figure 1.

### Evaluation of the functional coherence measure

**Figure 2:**
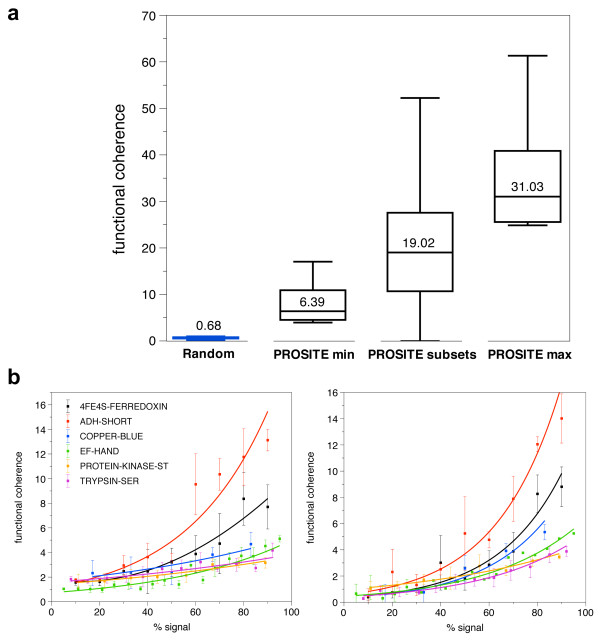
**Functional coherence of random, functional, and dilute functional clusters**. a) We show median functional coherence scores for random clusters, as well as clusters derived from functional site patterns. "PROSITE min" refers to the minimum cluster size for each PROSITE pattern cluster in Table 1 (derived from training sets used for existing FEATURE models [16]), while "PROSITE max" refers to the maximum size of each cluster. The PROSITE subsets were randomly sampled from the max PROSITE clusters, while the random clusters were randomly sampled from all Swiss-Prot proteins. The median functional coherence for the random clusters is clearly much lower than that for clusters derived from PROSITE. b) We plotted functional coherence as a function of percent signal. We decreased functional signal by randomly replacing members of the six "PROSITE min" clusters with either structurally similar proteins (left), or random proteins (right). Functional coherence decreases exponentially as the proportion of biological signal decreases.

In order to determine the suitability of the functional coherence measure for protein clusters, we compared the functional coherence of random protein clusters and clusters associated with six PROSITE patterns (Table 1). The functional clusters ranged in size from six proteins to over 1300 proteins. Functional clusters attain much higher functional coherence scores than random clusters, with median values of 6.39, 19.02, and 31.03 for the PROSITE min, PROSITE subsets, and PROSITE max functional clusters, respectively, and 0.68 for random clusters (see Figure 2a).

**Table 1:** Test clusters for evaluating functional coherence

PROSITE pattern	min # proteins	max # proteins
COPPER_BLUE	6	61
PROTEIN_KINASE_ST	9	1303
ADH_SHORT	10	262
4FE4S_FERREDOXIN	11	169
TRYPSIN_SER	13	399
EF_HAND	19	1248

We tested the functional coherence measure using clusters associated with six functional motifs from PROSITE. The minimum number of proteins is the smallest cluster size we used for that particular PROSITE pattern, derived from training sets from existing FEATURE models. The maximum number is the total number of proteins in Swiss-Prot annotated to that pattern.

We also calculated the functional coherence of the diluted clusters to see how the amount of signal in a cluster affects its functional coherence (see Figure 2b). Functional coherence clearly decreases as % signal decreases; when the size of the cluster is fixed, the relationship is approximately exponential. Note that the slight increase in functional coherence at very low % signal in the additive dilution sets is due to sharp increases in cluster size; the increase is negligible at the cluster sizes we worked with. Based on these observations, we set an empirical cutoff at 3.0 to distinguish functionally coherent clusters from non-functional clusters.

### Application of two-phase clustering to CYS microenvironments

We applied our two-phase clustering strategy to a data set of 19,253 FEATURE microenvironments centered on cysteine residues. The procedure is described in detail in the Methods section; briefly, we reduced the microenvironment vectors using principal component analysis and clustered them using *k*-means (*k *= 40 determined from a preliminary parameter search), and then applied hierarchical clustering-based cluster selection to each of these coarse-grained clusters. All clusters with at least five microenvironments were considered for further analysis, since five is the minimum number of sites we have used for training FEATURE models in the past. The cluster selection produced 218 clusters with more than five microenvironments.

Using a functional coherence cutoff of 3.0, we identified 70 clusters to annotate in more detail (clusters are named according to their coarse cluster ID and hierarchical clustering node ID, *e.g*. Clust1-Sub1). We also investigated clusters with high internal correlation (as defined by the hierarchical clustering procedure) but low functional coherence. We annotated the 70 functionally coherent clusters automatically with information from the Swiss-Prot knowledgebase [29], PDB heteroatoms, and PubMed [30] abstracts, dividing the results into rediscoveries of known functional sites (Table 2), novel predictions for individual proteins (Table 3), and clusters representing potentially novel functional sites (Table 4). Information for all clusters is available online [31].

When we examined clusters with low functional coherence but high internal correlation, we found that many were associated with disulfide bonds, surface-exposed regions, or experimental artifacts such as alternate coordinates. Coarse clusters 9 and 26 seem to consist predominantly of these types of clusters. Upon examining clusters with higher functional coherence, however, we see that they have more functional themes. Coarse clusters 32 and 33 are enriched for zinc-binding, coarse clusters 22 and 23 are heavily annotated with cytochromes, and coarse cluster 30 contains iron-binding clusters. Although the cytochrome-associated clusters are only found in coarse clusters 22 and 23, clusters related to metal ion-binding, phosphatase, and kinase activity are found across multiple coarse clusters.

**Table 2:** Rediscovered functional sites

cluster ID	Size	FC	Function
Clust1-Sub13	5	16.93	Copper binding, multicopper oxidase type with C1H2 coordination
Clust1-Sub52	7	3.73	Zinc binding, C2H2 and multi-HIS type
Clust1-Sub53	13	11.26	Zinc binding, 1 CYS + multi-HIS + ASP/GLU + H_2_O coordination, with several sites being dinuclear.
Clust1-Sub118	10	3.11	Zinc binding, C3H1 type
Clust1-Sub257	7	10.65	Associated with TYR phosphatases and adjacent to active site

Clust10-Sub26	7	7.36	Metal binding with four sulfur coordination - iron (2FE2S) with 2 CYS and zinc binding with 4 CYS

Clust21-Sub5	5	3.15	Tyrosine phosphatase active site
Clust21-Sub17	5	4.45	Iron binding, 2FE2S with additional CYS present
Clust21-Sub27	7	11.86	Tyrosine phosphatase active site, enriched for polyfunctional proteins

Clust22-Sub159	5	3.11	Iron binding, 4FE4S type with additional LYS and PRO nearby

Clust23-Sub44	10	15.20	Cytochrome C heme binding, C2H2 type
Clust23-Sub46	17	18.53	Cytochrome C heme binding, high molecular weight cytochromes
Clust23-Sub80	5	13.77	Cytochrome C heme binding, C2H2 type
Clust23-Sub83	7	3.15	Cytochrome C heme binding, additional CYS, MET, or LYS, 1 HIS, and at least 1 PRO present

Clust29-Sub110	6	4.65	Zinc binding, C3H1 type

Clust30-Sub15	6	4.45	Iron binding, 2FE2S oxidoreductase type with 3-4 CYS present
Clust30-Sub24	6	4.45	Iron binding, 2FE2S oxidoreductase type with 4 CYS
Clust30-Sub57	5	14.86	Iron binding, 2FE2S ferredoxin type with 3-4 CYS
Clust30-Sub110	6	12.81	Iron binding, 2FE2S ferredoxin type with 4-5 CYS
Clust30-Sub122	5	15.77	Iron binding, 2FE2S ferredoxin type with 4-5 CYS
Clust30-Sub160	10	24.48	Iron binding, mixed 2FE2S and 4FE4S with 4-6 CYS or MET

Clust31-Sub14	9	5.03	Ser/Thr protein kinase associated site corresponding to domain IX, adjacent to substrate recognition site

Clust32-Sub46	7	3.78	Zinc binding, multinuclear site (3-4) with 7+ CYS
Clust32-Sub62	5	4.51	Zinc binding with 4 CYS
Clust32-Sub208	6	3.03	Zinc binding with 4 CYS
Clust32-Sub222	15	14.95	Metal binding (zinc, iron) with 4 CYS
Clust32-Sub382	7	5.87	Zinc binding with 4 CYS and additional ASP and GLU nearby

Clust33-Sub49	6	17.69	Copper binding, blue copper C2H2 type
Clust33-Sub60	16	3.71	Zinc binding, mixed C2H2 and C3H1 type
Clust33-Sub63	5	4.78	Zinc binding, mixed C2H2 and C3H1 type
Clust33-Sub83	17	3.82	Zinc binding, majority C2H2 type, C3H1 have additional HIS nearby
Clust33-Sub99	13	3.65	Metal binding, iron has C1H4 type, zinc has C2H2 type with additional HIS nearby
Clust33-Sub109	8	6.13	Zinc binding, C3H1 type
Clust33-Sub156	6	4.29	Zinc binding, C2H2 type
Clust33-Sub237	10	6.26	Zinc binding, C3H1 type
Clust33-Sub343	6	4.44	Zinc binding, C3H1 type

These clusters represent functional annotations that are already known in that all or the vast majority of sites are annotated for that function. FC = functional coherence.

### Rediscovery of known sites

We identified many examples of known functional sites, including copper binding sites, tryosine phosphatase active sites, and a motif associated with Ser/Thr protein kinases. We present our observations on two of these functional sites, copper binding and zinc binding, in more detail below.

#### Copper-binding proteins

**Figure 3:**
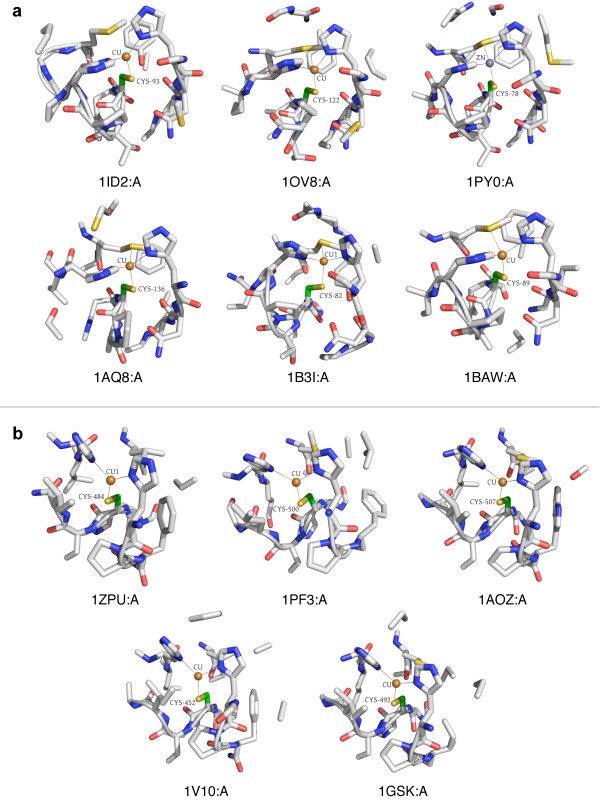
**Two distinct clusters for copper binding**. (a) Clust33-Sub49 consists of copper-binding environments from blue copper proteins involved in electron transport. (b) Clust1-Sub13 consists of copper-binding environments from multicopper oxidase proteins, so named because they contain multiple copper centers. The mode of binding for both types of proteins is similar. All microenvironment images were generated using PyMol [59].

Clust33-Sub49 represents a copper-binding site, with the majority of its member proteins belonging to the blue copper family of cyanins (see Figure 3a). One of the structures is bound to zinc rather than copper, but is known to bind copper in that location. All other structures in Clust33-Sub49 are bound to copper.

The microenvironment contains two HIS residues helping to coordinate the ion, and a MET residue, which is not always bound but is always nearby. Terms associated with copper-binding and electron transport dominate annotations for this cluster.

Another copper-binding cluster (Clust1-Sub13, see Figure 3b) is in an entirely different coarse cluster, and this environment seems to be associated with the family of multicopper oxidases. Again, all structures are bound to copper through the central CYS residue, in addition to two HIS residues. In three out of the five microenvironments, a MET residue is present but not bound. The annotations center around copper-binding, but with keywords for "oxidoreductase" rather than "electron transport", distinguishing the function of this cluster from that of Clust33-Sub49. Interestingly, both of these copper-binding clusters correspond to the same type of copper center - type 1, which is coordinated by CYS, two HIS residues and a fourth residue [32]. In plastocyanins, the fourth residue is a MET, while in multicopper oxidases it is often substituted by a non-coordinating residue [33]. This is consistent with our observations in these two clusters.

#### Zinc-binding clusters

**Figure 4:**
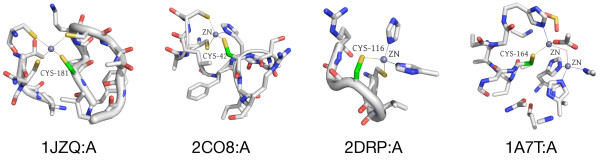
**Different types of zinc binding sites**. Our cluster selection approach divides several clusters into smaller groups of zinc binding site environments. Many of these represent different types of zinc binding sites: (from left to right) coordination by four CYS residues, coordination by three CYS and one HIS residue, coordination by two CYS and two HIS residues (C2H2 type), and coordination of multiple zinc ions by many diverse residues, including CYS, HIS, ASP, GLU, and water.

We also identified clusters representing conserved environments in protein kinases and cytochrome C proteins, as well as iron, iron-sulfur, and zinc binding sites. Zinc binding is particularly interesting, as there are many motifs and catalytic sites known to bind zinc [34]. Figure 4 shows four types of zinc binding sites present in distinct coarse clusters in our data set. The first three types are mononuclear, where a single zinc ion is coordinated by different numbers of CYS and HIS residues - 4 CYS, 3 CYS and 1 HIS, or 2 CYS and 2 HIS. Zinc-binding of this type is typically for protein structural stability. The fourth type shown is a cocatalytic dinuclear zinc site coordinated by a more diverse set of residues, including HIS, ASP, CYS, and water. These types of sites are found in metalloenzyme active sites, where the zinc ion is required for catalytic activity [35].

Since cysteine residues are often involved in binding metal ions, it is not surprising to see many clusters with metal-binding as the dominant functional annotation. We were, however, intrigued by the fact that many - such as zinc - did not group into the same coarse cluster. To investigate whether k-means was partitioning the clusters inappropriately, we combined 15 zinc-binding-associated clusters belonging to four coarse clusters into one large cluster and ran it through the cluster selection process again. The exact same clusters were produced (excluding two microenvironments from one cluster that were deemed singletons in the new result), indicating that the cluster boundaries from k -means are robust.

Although many of the zinc-binding clusters differ according to the number and type of amino acids that coordinate the zinc, several disparate clusters do bind zinc in a similar manner. When we examined the sets of principal component vectors for clusters with identical coordination types, we confirmed that there are indeed significant differences between them. Therefore, while the coordinating residues are identical, there are apparently subtle ways - specific principal components - in which they differ.

As each principal component is composed of weighted versions of the original microenvironment features, we can deduce which features contributed most heavily to these differences. For the four clusters that bind zinc using four cysteines, features corresponding to arginine, histidine, and non-canonical residues vary significantly, as do features related to aromatic and aliphatic carbons and amide nitrogens. The five clusters binding zinc using three cysteines and one histidine differed in features related to VAL, PHE, CYS, and THR residues, as well as aromatic and aliphatic carbons, hydroxyl and carboxyl oxygens, and charge. The two C2H2 clusters also showed distinctions in features related to aromatic atoms, as well as amide atoms, different types of charges, and LEU and HIS residues. See Additional files 1, 2 and 3 for more details on the zinc cluster analysis.

### Predictions of functional sites

**Figure 5:**
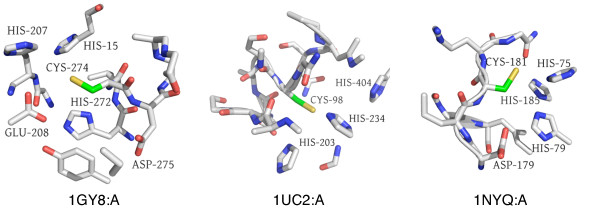
**Potentially novel zinc binding sites in Clust1-Sub53**. We predict zinc binding sites for (from left to right) structures 1GY8:A (no Swiss-Prot accession number) at CYS274, 1UC2:A [Swiss-Prot:O59245] at CYS98, and 1NYQ:A [Swiss-Prot:Q8NW68] at CYS181 based on zinc binding for other microenvironments in this cluster. Features supporting this prediction include the presence of multiple HIS residues and occasionally ASP or GLU, all known to coordinate zinc.

We identified several microenvironments that may be novel metal-binding sites, some of which have supporting evidence or predictions made by other methods (see Table 3). Several proteins in Clust1-Sub53, a zinc-binding cluster, have not been proven to bind zinc at the sites specified (CYS181 in 1NYQ:A [PDB:1NYQ], CYS98 in 1UC2:A [PDB:1UC2], and CYS274 in 1GY8:A [PDB:1GY8]). Their microenvironments are highly suggestive of zinc binding, with the salient features being the presence of several HIS residues and occasionally an ASP or GLU residue around the central CYS (see Figure 5).

**Table 3:** Novel annotations for individual proteins

PDB ID	Site residue	cluster ID	Annotation
1GY8:A	CYS274	Clust1-Sub53	Zinc binding
1UC2:A	CYS98	Clust1-Sub53	Zinc binding
1NYQ:A	CYS181	Clust1-Sub53	Zinc binding
1OKG:A	CYS278	Clust22-Sub159	Iron binding

These annotations represent predictions for proteins in clusters where the function can be readily identified. The functional coherence for Clust1-Sub53 is 11.26 and functional coherence for Clust22-Sub159 is 3.11.

There is evidence that 1NYQ and 1UC2 may bind zinc at those locations. Others have noted the presence of conserved HIS and CYS residues in 1UC2 corresponding to those in our site, similar to zinc metalloenzymes and tRNA synthetases [36]. Goyal and Mande [37] also predicted metal-binding at the same location using a structural template method. 1NYQ, a threonyl-tRNA synthetase, is already known to bind zinc [38], but in the crystal structure zinc is bound at a location far from our site. CYS181 may thus be a novel zinc binding site for 1NYQ. The third protein, 1GY8, is a UDP-galactose 4'-epimerase from *T. brucei *[39] that is not known or suspected to bind zinc.

### Clusters with potentially novel biological significance

There are many clusters with more obscure themes that may have some biological importance (listed in Table 4). We describe several examples in more detail below.

**Table 4:** Potentially novel functional sites

cluster ID	Size	FC	Putative annotation	Distinguishing features
Clust4-Sub23	5*	8.79	Structural role	Extended beta sheet environment with repeated CYS flanked by PHE. * Several sites are adjacent to one another, and may be involved in disulfide bonds.
Clust5-Sub70	12	3.07	TYR phosphorylation site, possibly autocatalytic	2/3 of proteins are TYR kinases with multiple phosphorylation sites. Environment characterized by loop containing CYS, MET, and TYR.
Clust6-Sub240	5	4.33	Associated with ligand binding	80% of sites are near a bound ligand.
Clust8-Sub25	11	4.12	Structural role	Inward facing CYS on a surface accessible helix surrounded by an abundance of aliphatic, hydrophobic sidechains.
Clust8-Sub352	6	4.18	Structural role	Helical CYS in the vicinity of 1 HIS and several aliphatic, hydrophobic sidechains.
Clust15-Sub152	6	4.43	Associated with enzymatic activity	All proteins are enzymes. Environment contains multiple ARG and occasionally HIS.
Clust21-Sub48	9*	3.06	Associated with WD repeat motif	Environment characterized by beta sheets and the presence of another CYS. * Several sites are adjacent to one another.
Clust24-Sub17	5	4.34	Functional role	Environment contains an ASP, a GLU, and usually at least one LYS, all charged and polar residues.
Clust25-Sub19	5	6.32	Associated with sugar kinases	Beta sheet environment with multiple sulfur- containing residues.
Clust31-Sub18	5	7.00	Protein binding	80% of proteins are protein-binding. Environment characterized by helical CYS and an opposing TRP residue.
Clust36-Sub127	5	5.04	Functional role	Environment is solvent exposed with an ASP and LYS forming a possible triad with the CYS.
Clust39-Sub58	5	18.34	Associated with viral proteins	Sparse environment containing TRP, TYR, THR, and ARG, all polar and mostly hydrophobic residues.

These predictions represent clusters where the function is not obvious but reasonable evidence exists for a coherent functional theme, even if it is in a structural role. FC = functional coherence.

#### A recurrent CYS hydrophobic helical motif

**Figure 6:**
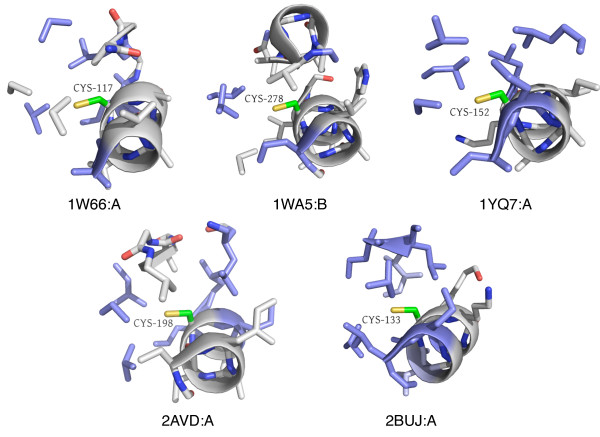
**Clust8-Sub25 - Novel microenvironment motif with a potential structural role**. Five representative microenvironments from a total of 11 are shown. This set of microenvironments is characterized by the central CYS based in a helix with the sidechain surrounded by an abundance of aliphatic, hydrophobic sidechains (ILE, LEU, VAL). Cysteines are often important for stabilizing protein structures, and the absence of reactive sidechains combined with the striking similarity between members of this cluster suggest a potential structural role for this microenvironment.

Clust8-Sub25 (see Figure 6) has 11 microenvironments, all of which are characterized by an alpha helix containing the central CYS residue, whose sidechain is surrounded by an abundance of hydrophobic, aliphatic residues such as isoleucine, leucine, and valine. The residues present in the microenvironment are discontinuous in sequence and structure, making it unlikely that other methods would be able to identify it.

Since the microenvironment is not usually surface-exposed, it may be reasonable to exclude explicit functions such as catalysis or binding to small molecules. Interestingly, all but three microenvironments (in 1W66:A, 1L1Q:A, and 1VRW:A) are involved in leucine-rich hydrophobic interfaces within helix bundles. Buried CYS residues typically participate in disulfide bridges and so the fact that these microenvironments consist of a solitary buried CYS in a hydrophobic context is interesting and raises the possibility that it is playing more than a structural role. There is, in fact, precedence for leucine-rich environments being involved in protein-protein binding with a cysteine residue potentially regulating the interaction [40].

#### A potential phosphorylation motif

**Figure 7:**
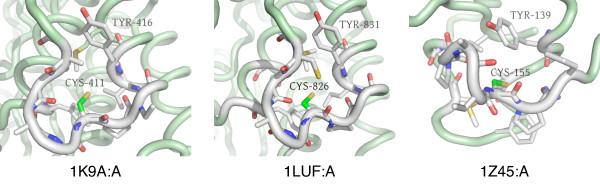
**Clust5-Sub70 - potential TYR autophosphorylation site**. This cluster contains 12 microenvironments, eight of which belong to tyrosine kinases. In the eight kinase microenvironments, the CYS is on a loop next to a helix containing a TYR residue; the environment as a whole is surface-exposed and contains additional sulfur-containing residues. From left to right, we show 1K9A:A [Swiss-Prot:P32577], in which TYR416 is annotated as a putative autophosphorylation site (by similarity), 1LUF:A [Swiss-Prot:Q62838], in which TYR831 is not annotated as a potential phosphorylation site, and 1Z45:A [Swiss-Prot:P04397], a yeast aldose 1-epimerase, which is not a TYR kinase. There is, however, a surface-exposed TYR in a loop environment with an additional sulfur-containing residue.

Another intriguing example is Clust5-Sub70 (see Figure 7). This cluster contains 12 microenvironments, eight of which are from protein tyrosine kinases. The site, however, does not correspond to the active site, but to a surface-exposed loop. In the kinases and in one of the other four sites, a yeast aldose 1-epimerase, there is a tyrosine residue within or adjacent to the microenvironment. One or two other sulfur-containing sidechains are also present. Since the kinases are all known to be phosphorylated, it is possible that the tyrosine in the microenvironment may represent a phosphorylation site. In fact, one of these tyrosines, TYR416 in 1K9A:A, is annotated in Swiss-Prot as a putative autophosphorylation site [Swiss-Prot:P32577]. The other kinase-associated sites are not annotated, but it is conceivable that they may also be phosphorylation sites, perhaps by autophosphorylation. The other four microenvironments belong to two aminotransferases, an epimerase, and a viral coat protein, and the significance for these cases is not clear.

#### A solvent-exposed D-C-K surface motif

**Figure 8:**
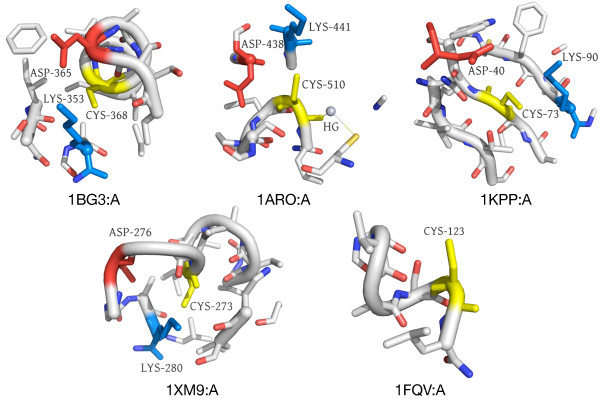
**Clust36-Sub127 - Novel microenvironment motif with a potential functional role**. This microenvironment motif is surface exposed and contains an ASP (red) and a LYS (blue) around the central CYS (yellow) in a potentially functional triad in four out of five cases. As these are all residues known to participate in chemical reactions, it is possible there is an active role for this recurring microenvironment.

Lastly, we present Clust36-Sub127, a set of five surface-exposed microenvironments (see Figure 8). The microenvironments from this cluster come from five proteins: Plakophilin-1 [Swiss-Prot:Q13835], involved in epidermal morphogenesis; Tumor susceptibility gene 101 protein [Swiss-Prot:Q99816], part of a vesicular trafficking complex; S-phase kinase-associated protein 2 [Swiss-Prot:Q13309], the substrate recognition component of a protein degradation complex; a DNA-directed RNA polymerase [Swiss-Prot:P00573]; and Hexokinase-1 [Swiss-Prot:P05708], involved in carbohydrate metabolism. The proteins are diverse in function and come from evolutionarily diverse organisms. Four out of the five are annotated as phosphoproteins. A striking feature, however, is the presence of a solvent-exposed ASP-CYS-LYS motif, where the CYS represents the center of the microenvironment.

As all of these residues are capable of being catalytically active, their solvent accessibility and placement together make it possible that this microenvironment could play a functional role. Some classes of enzymes utilize a CYS-HIS-ASP catalytic triad; for certain transferases it is posited that the HIS is not critical for catalytic activity [41]. It is thus possible that the main functional elements are the CYS and ASP residues. As both LYS and HIS have basic sidechains, it is also possible that LYS could perform a similar role as HIS in this context. Additional studies have identified conserved CYS-GLU-LYS triads in the nitrilase superfamily [42] and a similar triad in an amidase from *P. aeruginosa *[43]. GLU and ASP are very similar amino acids and often perform analogous functional roles. Although these known sites tend to be found in catalytic clefts, they provide some support for this microenvironment's potential functional significance.

### Cluster annotation output

**Figure 9:**
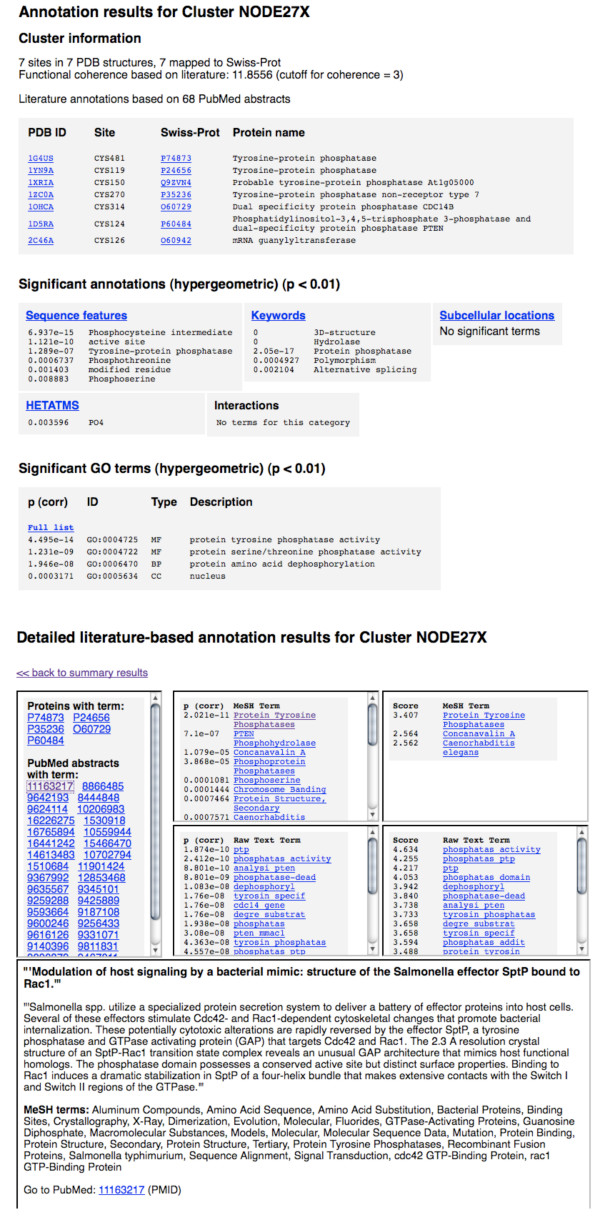
**Example annotation output for Clust21-Sub27, TYR phosphatase active sites**. The HTML output for the cluster annotation method is shown for a tyrosine phosphatase active site cluster. A summary page showing general cluster information and top significant terms for each annotation type contains links to more detailed information for each type of annotation, including lists of proteins mapped to each annotation term. Detailed literature output shows the proteins and PMIDs contributing to each annotation term and abstract text for each PMID.

Each cluster that scored 3.0 or more for functional coherence was analyzed for enriched terms from Swiss-Prot, PDB HETATMs, and PubMed abstracts as described in the Methods. Summary HTML pages with lists of the top-ranked terms contain links to more detailed pages showing contributions to each term by Swiss-Prot, PDB, or PubMed ID. There are also external links to the source databases for each protein, HETATM, and literature abstract. See Figure 9 for sample screen shots. The annotation output for each cluster in Tables 2, 3 and 4 is available online at http://feature.stanford.edu/clustering/cys[31].

## Discussion

Protein function prediction has traditionally concentrated on modeling known functional domains and motifs. This becomes a problem when increasing numbers of newly discovered proteins lack similarity to existing ones and when the new proteins may contain novel biological functions. In this work, we present a two-step unsupervised clustering procedure combined with automated knowledge-based characterization methods - a pipeline that can be applied over a large number of protein structures to discover potentially novel functional sites systematically.

This pipeline uses a site representation - the FEATURE microenvironment - that is sequence independent and captures physicochemical properties in a radially averaged way. Although detailed geometric information is not retained, the representation is robust to differences in exact sequence or structure. As shown in previous work [16], this allows more robust detection of function in proteins that are evolutionarily more distant.

In defining optimal clusters in the hierarchical clustering step, we use both internal and external coherence measures. Node correlation gives an indication of how physically similar two sets of microenvironments are, while the functional coherence measure approximates the biological signal through shared literature connections. This enables us to define a cutoff above which we consider a cluster for further investigation.

We have applied this pipeline to microenvironments centered on cysteine residues. The resulting clusters recapitulate many well-known cysteine-related functions, such as metal-binding and tyrosine phosphatase activity; these clusters can easily be used to train supervised FEATURE models. In addition, we make three kinds of novel predictions: new annotations for individual proteins, new 3D motifs with putative function, and new 3D motifs with unknown function. In particular, we predict novel zinc binding sites in structures 1NYQ, 1UC2, and 1GY8; a potential tyrosine phosphorylation motif; an aliphatic microenvironment motif that may play a structural role; and a microenvironment motif containing a potentially active residue triad.

### A two-phase clustering approach allows for fewer initial assumptions

One of the drawbacks of *k*-means clustering is that we must specify the number of clusters beforehand. Although heuristics can provide reasonable estimates, it is still a challenge to set parameters when the true structure of the data is not known *a priori*. In this work, we demonstrate a two-step approach that would allow for fewer assumptions in the initial clustering and provide better separation in subsequent analyses. The ability to post-process large "coarse" clusters into smaller, more coherent clusters means that we do not have to attempt to divide all the objects into the most optimal groups at the outset, but can simply group them into coarse "ballparks". We can then use more accurate but more expensive methods such as hierarchical clustering to identify finer-grained distinctions within the large groups.

Our analysis of the zinc-binding clusters illustrates this point. If we had had prior knowledge of these clusters, we may have attempted to constrain the *k*-means to produce a cluster with all of these. The hierarchical clustering, however, picked out biologically reasonable clusters while freeing us from attempting to deduce the underlying structure of the data in the *k*-means step. The boundaries between the zinc-binding clusters themselves were robust, as shown by combining and re-clustering.

### Using different coherence measures allows for flexibility in cluster selection

By adapting the method from Raychaudhuri *et al *[44], we select clusters from a hierarchical clustering to strike a balance between internal and external coherence. Higher internal coherence indicates that the microenvironments are physically similar, while higher functional coherence suggests better supporting evidence in literature and knowledgebases for the possible biological causes of the similarity. We use a scoring function that is approximately equal in its weighting of these two coherence measures, but the function can be easily modified to suit particular needs.

To recapture only well-known functional sites, a function heavily weighted towards functional coherence would perform better. Weighting the function more towards internal coherence would produce clusters that are very physically similar, but may not have clear or meaningful biological significance. In fact, many of the clusters may instead be artifactual or strictly structural, reflecting, for instance, ambiguously defined coordinates or disulfide bonds.

Our method is flexible in that we can modify the scoring function to make different types of discoveries. Clusters that are already well characterized (as suggested by high functional coherence) may have one or two members that are not annotated with that particular function; we can then transfer the annotation indicated by the cluster analysis to those members. Clusters that emphasize internal coherence but have low functional coherence, on the other hand, may sometimes represent completely novel functional sites. Somewhere in between lies a third type of discovery - that of a 3-dimensional motif for a characterized function that did not previously have a defined motif. These three types of discoveries could be described as "individual protein annotation", "motif identification", and "novel biological site", and each one is more difficult to validate than the former. Each type is, however, also more interesting from a discovery standpoint than the preceding type.

### The approach identifies recurring microenvironments

Through rediscovery of metal binding sites and active sites for tyrosine phosphatases, we demonstrate that this method is effective at identifying functionally important environments; further analysis of the zinc and copper binding examples shows that it can also be precise, distinguishing between different types of binding sites for the same ligand. Several of our novel predictions for individual binding sites can be corroborated by other methods or evidence in the literature, though there are also a few that are more novel. 1NYQ is not known to bind zinc at the location we predict, but it does have other zinc binding sites, and the presence of multiple binding sites in the same protein is not uncommon. The available knowledge for 1GY8 makes no indication of zinc binding activity.

More interesting are the clusters that have no readily apparent functional theme according to known database annotations and literature and yet have very similar physicochemical microenvironments. In some cases, they correspond to distinct substructures, such as a repeating beta sheet motif. This type of motif is known to make up entire domains in some cases, such as the pectin lyase-like fold [45], which we find in Clust4-Sub23, or may be repeated in different numbers on a smaller scale as with WD-repeats [46], which we find in Clust21-Sub48. In other cases, the environment does not correspond to a known structural motif; indeed, in the majority of cases, the residues involved are highly discontinuous in sequence.

Although these are difficult to validate, they are also the most intriguing. Some may be functional regions involved in interaction with other proteins or molecules (*e.g*. Clust36-Sub127); others may be involved in structural stability (*e.g*. Clust8-Sub25) or represent a remnant of shared evolutionary history. When the microenvironments come from diverse proteins it becomes more likely that they demonstrate examples of convergent evolution on a small scale or the re-use of chemically and physically favorable elements -potential modular building blocks [47]. Indeed, protein domains may be defined as a combination of recurring substructures [48], and the view that smaller units are important for function is supported by many studies [21,47,49].

Other tools exist which view protein structure space as consisting of sub-substructures or fragments, such as Fragnostic [50]. The use of microenvironments here provides a unique approach to this problem for it captures chemical properties of amino acids, which are major contributors to biological function, without constraining linearity in the sequence or structure. We also do not assume any evolutionary relationship. At lower levels of similarity, we may be able to detect environments that recur despite global differences, and environments that have converged from different origins. Recurring microenvironments may also reveal general principles for protein stability and function.

### Clustering is a useful tool for exploration and hypothesis generation

Beyond recapitulating known functions and identifying potentially novel sites, our cluster analysis approach allows open-ended exploration of protein function space as described by microenvironments. The hierarchical trees that underlie the cluster selection process themselves have inherent value; we can use them to see how similar functional microenvironments are to each other, perhaps teasing out evolutionary relationships or the changes needed to convert a particular environment into another.

When testing parameters for hierarchical clustering, we used data sets consisting of known groups of microenvironments based on PROSITE motifs, but the resulting trees did not always map cleanly back to those groupings. Often, individual microenvironments were excluded either because their inclusion negatively impacted the internal or external coherence, or because they were located in a different area of the tree. Both cases suggest that the microenvironments for these "singleton" sites differ in some way from that of the other sites mapped to the same PROSITE motif. What makes these sites so different, and what implications does this have for their classification? What might this say about the evolution of a particular function?

Inspection of the overall tree of clusters can also lead to interesting questions, for we can see how the microenvironments of different functions relate to one another. For example, different types of protease active sites - including zinc proteases, serine proteases, and thiol proteases - have microenvironments that cluster distinctly into different areas of a hierarchical tree (see Additional file 4). These observations make sense given the diverse origins of proteases, many of which arose independently even while sharing very similar catalytic mechanisms [51,52]. Other dissimilarities - or similarities - between different classes of enzymes may be less well known and worth investigating.

## Conclusions

Structural genomics efforts are rapidly expanding the diversity of known protein structures. The lack of functional annotation for many novel structures and the identification of novel biological functions are two problems that existing methods have yet to address. We have developed an unsupervised approach for exploring protein structure-function space that identifies groups of recurring 3D protein microenvironments that can be discontinuous in sequence. By using a two-phase clustering approach, we incorporate flexibility into the procedure, while the addition of external knowledge enables better filtering and interpretation of resulting clusters. We applied this approach to a set of cysteine microenvironments, identifying many known functional site motifs as well as novel predictions for individual proteins and potentially novel biological motifs. This approach can be applied across other amino acid data sets to discover additional recurring microenvironments.

## Methods

The functional site discovery and annotation pipeline consists of four main steps: 1) generation of a large set of protein microenvironments; 2) coarse-grained clustering of the generated microenvironments; 3) fine-grained clustering of coarse clusters and cluster selection; and 4) cluster analysis and annotation. Figure 1 illustrates this pipeline. We describe each of the steps in more detail below.

### Microenvironment data set

We use the term "microenvironment" to refer to a local, spherical region in a protein structure that may encompass residues discontinuous in sequence and structure. Specifically, we use the FEATURE system to represent a microenvironment using a set of 44 physicochemical properties collected over six concentric spherical shells centered on a site of interest. The total radius of the microenvironment is 7.5 Å. This representation is described in Wei *et al *[12] and Halperin *et al *[17].

We generated microenvironments for cysteine (CYS) residues derived from the previously published study by Yoon *et al *[25], filtered to remove most cysteines participating in disulfide bonds. This resulted in 19,253 CYS-centered microenvironments. All microenvironment vectors were normalized by the standard deviation of each feature across the entire set of vectors.

### k-means clustering

We used *k*-means clustering to achieve a coarse-grained clustering of the set of CYS microenvironments obtained above. The general algorithm is described in detail elsewhere [53]. Preliminary analysis of a range of values for *k *showed that *k *= 40 with vectors reduced to 80 principal components provided the best correspondence to known PROSITE motifs (see Additional file 5). We used cosine similarity as the distance metric to compute distances between vectors and cluster centers.

### Evaluation of functional coherence

We needed a method to assess the biological coherence of a protein cluster given no information about functional class labels. A method developed previously, called neighbor divergence per gene (NDPG) [27,28], adapts well to this purpose. NDPG calculates a measure called functional coherence, which is based on shared, similar literature between cluster genes. NDPG requires a mapping between genes (proteins, in our case) and documents, and a list of semantic neighbors for each document. We mined mappings between proteins and PubMed identifiers (PMIDs) from Swiss-Prot (version 55.4), filtering out publications labeled as "large scale" studies. Our documents were thus PubMed abstracts, which we represented as weighted word vectors and compared using cosine similarity as described by Raychaudhuri *et al *[27]. The 20 most similar documents to a target document are considered its semantic neighbors.

For a protein cluster, then, we compute a score for each document mapped to each protein as the sum of the fractional references of that document's semantic neighbors. The fractional reference is the proportion of proteins mapped to a document that are present in the given cluster. The document scores for each protein forms an observed distribution, which is compared to a theoretical Poisson distribution using Kullback-Leibler (KL) divergence. We then average the KL divergence over all proteins in the cluster to produce a functional coherence measure. The algorithm is described in more detail elsewhere [27,28].

To evaluate the behavior of the functional coherence measure, we devised a number of test clusters. We constructed six clusters (designated as PROSITE min) varying in size from 6 to 19 proteins with each cluster corresponding to a PROSITE pattern. All of the proteins have structures in the PDB and less than 50% sequence identity to other proteins in the same cluster. These clusters are thus similar to that which we might obtain from a clustering of PDB microenvironments, with 100% biological signal, and should have very high functional coherence scores.

We also diluted the signal in each cluster by adding proteins (additive) or replacing proteins (fixed) with random or structurally similar proteins. The structurally similar proteins were obtained using the tool S-BLEST [54,55]. The diluted clusters allowed us to see how the amount of biological signal in the cluster - as represented by the proportion of the cluster corresponding to the original proteins - affected functional coherence. In addition, we retrieved the full set of Swiss-Prot records associated with each PROSITE pattern (designated as PROSITE max) and sampled randomly from them to construct 600 total clusters (100 for each of the patterns) varying in size from 10 to about 1400 proteins (designated as PROSITE subsets). We also created 600 random clusters varying in size from 10 to 1400 proteins. We calculated the functional coherence for each set of clusters.

### Hierarchical clustering and cluster selection

The *k*-means clustering is employed as a coarse-grained partitioning step in our approach. Agglomerative hierarchical clustering [53] merges the most similar pairs of items successively until all items have been joined, producing a binary tree. Each node in the tree represents a possible cluster composed of the items descended from that node. We apply hierarchical clustering to each coarse cluster obtained in the *k*-means step and evaluate possible clusters using a combination of internal and external coherence measures.

We use Cluster 3.0 software [56] to perform agglomerative hierarchical clustering. Parameter testing using a test set mapped to known PROSITE motifs indicated that cosine similarity as a distance metric with single linkage as the linkage method produced clusters with the highest precision (external evaluation measure) and silhouette widths (internal evaluation measure) (see Additional file 6). For cluster selection, internal coherence for each cluster is taken as the correlation between the two branches at the corresponding node in the hierarchical tree as computed by Cluster 3.0. External coherence is represented using functional coherence.

Because we are interested in identifying clusters that are both internally consistent and externally meaningful, we score each node in the hierarchical tree with the following scoring function, which takes into account the node correlation (C), functional coherence (F), and the cluster size (N):

Node correlation varies from 0 to 1 and functional coherence is real-valued and greater than zero. If the score at a node is greater than the sum of scores at selected descendant nodes, that node becomes selected and its descendants are all deselected. Thus, the process outputs a disjoint set of nodes representing optimal, non-overlapping clusters. The algorithm is described in more detail elsewhere [44].

We applied the cluster selection procedure to the 40 coarse clusters produced by *k*-means, limiting the output to clusters containing at least five microenvironments. We then further filtered the resulting clusters to have functional coherence scores greater than 3.0, which we empirically determined to be an appropriate cutoff for distinguishing functional clusters from random clusters.

### Cluster analysis and annotation

We use information from multiple sources, namely PubMed abstracts, Swiss-Prot protein records, and PDB data files, to identify potentially useful terms to describe our protein clusters. Terms and mappings between proteins and PMIDs were derived from version 56.9 of Swiss-Prot, released in March 2009, containing 412,525 protein records. Note that Swiss-Prot is the manually reviewed portion of the larger Uniprot database, and we do not consider records from the unreviewed TrEMBL database. We then downloaded abstracts from PubMed based on the PMIDs extracted from the Swiss-Prot records, filtering out those labeled as "large scale" studies. We also used information from PDB entries in the non-redundant set of structures. We downloaded data from all three databases as XML and used the Python lxml[57] package to process it for the desired terms.

¿From the PDB files, we extracted labeled ligands (HETATMs) associated with each solved structure. From Swiss-Prot records, we extracted keywords, GO terms, sequence features such as binding sites, subcellular localization information, and protein-protein interactions, in addition to mappings to PMIDs. The annotations were stored only if they were determined experimentally or otherwise verified, excluding keywords, which do not have such labels. For each PubMed record, we extracted the manuscript titles and abstracts as raw text, and the Medical Subject Headings (MeSH terms) [58] associated with each PMID. The raw text is filtered for stop words and tokenized according to whitespace and punctuation, with hyphenated words treated as a single token. We consider both single tokens (unigrams) and consecutive pairs of tokens (bigrams) as terms.

#### Hypergeometric scoring

To produce ranked lists of terms of each type, we calculate a p-value for each term based on the hypergeometric distribution. This requires first collecting counts for each term in a category over the entire set of Swiss-Prot, PDB, or PubMed records, depending on the database from which the term was derived. Given a term, we then compute the p-value as follows:

To correct for multiple hypothesis testing, we multiply this p-value by the number of terms in that category. We use a corrected p-value cutoff of 0.01 for reporting significant terms in our annotation output.

#### Entropy-based scoring

In addition to the hypergeometric score, we devised an entropy-based scoring function for literature-based terms that takes into account the distribution of a term across abstracts as well as proteins. This scoring function rewards terms for which the responsible abstracts are more evenly distributed among the proteins in the cluster. The score for a term *t *is computed as follows:

where *D*_*tp *_is the ratio of the number of documents containing the given term in the given protein *p *to the number of documents containing the term in the entire cluster, and *idf*_*t *_is the inverse document frequency of the term - in other words, the relative significance of the term in the general corpus of abstracts. The score is normalized by the maximum score achieved over all terms in the cluster prior to weighting by *idf*_*t*_. Based on preliminary results using known functional clusters, we use an empirically derived score cutoff of 2.7 to identify useful terms. Empirical tests of variations of this scoring function showed that including additional components, such as the term frequency within documents and the fraction of proteins containing the term, did not improve results.

#### HTML-based output of annotation results

To facilitate exploration of the annotation results, we generate an HTML summary page displaying general information about the cluster along with the top ranked terms in each category. Links from this page lead to external databases (for PDB IDs, HETATMS, and Swiss-Prot accession numbers) or to more detailed pages showing all of the terms scored for each category and the lists of proteins that contributed to each term. The detailed literature annotation pages show the Swiss-Prot proteins and PMIDs associated with each top ranked term, and clicking on a PMID brings up the title and text for that abstract, with an external link to PubMed. See Figure 9 for sample screenshots of the output.

## Authors' contributions

SW performed evaluation of the functional coherence measure, implemented the cluster selection procedure, developed and implemented the cluster annotation methods, analyzed the data resulting from their application, and wrote the manuscript. TL performed analyses related to *k*-means, produced the coarse-grained clusters, and helped edit the manuscript. RBA guided the development of the methods and the interpretation of results, and helped edit the manuscript. All authors read and approved the final manuscript.

## Supplementary Material

Additional file 1 **Figure S1. Feature vectors from similar zinc-binding clusters**. We compared feature vectors from clusters corresponding to similar zinc-binding modes - 4CYS (top), 3CYS+1HIS (middle), and 2CYS+2HIS (bottom). The heat maps were generated from a hierarchical clustering of 15 zinc-binding clusters from 4 different coarse clusters. There clearly are major differences between even close clusters like Clust33-Sub60 and Clust33-Sub63. See Additional file 3 - Table S1 for lists of heavily weighted features contributing to the major principal components differing between clusters within similar zinc-binding modes. Click here for file

Additional file 2 **Figure S2. Hierarchical tree of zinc-binding clusters**. We combined the microenvironments from 15 zinc-binding clusters derived from 4 coarse clusters and repeated the cluster selection process. All of the original clusters were found again in the new output with the exception of two microenvironments which became singletons. The tree below (branch lengths not to scale) shows a conceptual ordering between clusters in the new clustering result, with new node labels within the node boxes and original labels below. The zinc binding mode is also indicated below each node (C4 = 4 CYS, C2H2 = 2 CYS + 2 HIS, C3H1 = 3 CYS + 1 HIS, and +H = an additional HIS in the environment not shown as coordinating the ion in the structure). The width of each node box represents the size of that cluster. Click here for file

Additional file 3 **Table S1. Top contributing features for principal components showing distinct differences between zinc-binding clusters**. We expect to see differences between clusters binding zinc with different residues, but many distinct clusters actually bind zinc in the same way. This table shows the features that contribute the most to the differences between distinct clusters binding zinc in the same way. Each column contains six principal components that showed the most variation between different clusters with the same type of zinc binding. For clusters binding zinc with 4 CYS, principal components 2, 10, 56, 54, 51, and 39 differed the most between them; for clusters binding zinc with 3 CYS and 1 HIS, components 10, 9, 71, 25, 49, and 2 differed the most; and for clusters binding zinc with 2 CYS and 2 HIS, components 2, 12, 54, 55, 39, and 7 differed the most. The top five most heavily weighted features for each principal component are shown with the feature name followed by its weight in that component. These observations demonstrate that even sub-types of zinc binding can be delineated further on the basis of less obvious features. Click here for file

Additional file 4 **Figure S3. Hierarchical tree of 15 known functional clusters**. We combined the microenvironments from 15 clusters representing training sets for FEATURE models and performed the cluster selection process. The results clearly separate out these 15 clusters (with the two clusters for Alcohol dehydrogenase corresponding to microenvironments centered on two points on the active site tyrosine). The relationships between the 15 clusters in the hierarchical tree may be interesting for further study. (Note: branch lengths are not to scale.) Click here for file

Additional file 5 **Figure S4. Parametrization for k-means**. To evaluate the optimal number of principal components and value for *k*, we calculated the average hypergeometric distribution probability of the best represented cluster across each of 21 PROSITE motifs enriched in the CYS data set for each set of parameters. Using 80 principal components with *k *= 40 resulted in the best performance while still allowing a reduction in the number of dimensions. Click here for file

Additional file 6 **Figure S5. Single linkage hierarchical clustering with cosine similarity distance metric results in better silhouette widths and precision**. We evaluated silhouette widths, purity (precision), and inverse purity (recall) of clusters produced from running the cluster selection process on a test set consisting of about 1400 microenvironments belonging to about 160 PROSITE patterns. We hierarchically clustered vectors corresponding to different numbers of principal components using both cosine similarity and Euclidean distance and using average, complete, or single linkage. (a) We plotted the distribution of silhouette widths resulting from each combination of parameters (blue = single linkage, red = complete linkage, black = average linkage). Single linkage and cosine similarity produce better silhouette distributions for all combinations. (b) We plotted the average inverse purity (recall) and purity (precision) for clusters resulting from each combination of parameters. Single linkage and cosine similarity produce clusters with higher purity. Click here for file

## References

[B1] Hendrickson WA Impact of structures from the Protein Structure Initiative Structure 2007 15 12 1528 1529 10.1016/j.str.2007.11.006 18073103

[B2] Lattman E The state of the Protein Structure Initiative Proteins 2004 54 4 611 615 10.1002/prot.20000 14997556

[B3] Brenner SE A tour of structural genomics Nat Rev Genet 2001 2 10 801 809 10.1038/35093574 11584296

[B4] Marsden RL Lewis TA Orengo CA Towards a comprehensive structural coverage of completed genomes: a structural genomics viewpoint BMC Bioinformatics 2007 8 86 1528 1529 10.1186/1471-2105-8-86 PMC1829165 17349043

[B5] Sonnhammer E Eddy S Birney E Bateman A Durbin R Pfam: multiple sequence alignments and HMM-profiles of protein domains Nucleic Acids Res 1998 26 320 322 10.1093/nar/26.1.320 9399864 PMC147209

[B6] Gough J Karplus K Hughey R Chothia C Assignment of homology to genome sequences using a library of hidden Markov models that represent all proteins of known structure J Mol Biol 2001 313 4 903 919 10.1006/jmbi.2001.5080 11697912

[B7] Hulo N Bairoch A Bulliard V Cerutti L De Castro E Langedijk-Genevaux P Pagni M Sigrist C The PROSITE database Nucleic Acids Res 2006 32 227 230 10.1093/nar/gkj063 PMC1347426 16381852

[B8] Attwood TK The PRINTS database: A resource for identification of protein families Brief Bioinform 2002 3 3 252 263 10.1093/bib/3.3.252 12230034

[B9] Holm L Sander C The Dali/FSSP classification of three-dimensional protein folds Nucleic Acids Res 1997 25 231 234 10.1093/nar/25.1.231 9016542 PMC146389

[B10] Krissinel E Henrick K Secondary-structure matching (SSM), a new tool for fast protein structure alignment in three dimensions Acta Cryst 2004 D12 2256 2268 10.1107/S0907444904026460 15572779

[B11] Laskowski RA Watson JD Thornton JM Protein function prediction using local 3D templates J Mol Biol 2005 351 614 626 10.1016/j.jmb.2005.05.067 16019027

[B12] Wei L Altman RB Recognizing protein binding sites using statistical descriptions of their 3D environments Pac Symp Biocomp 1998 497 508 9697207

[B13] Marsden RL Orengo CA Target selection for structural genomics: an overview Methods Mol Biol 2008 426 3 25 full_text 18542854 10.1007/978-1-60327-058-8_1

[B14] Chothia C Lesk AM The relation betwen the divergence of sequence and structure in proteins EMBO J 1986 5 823 826 3709526 10.1002/j.1460-2075.1986.tb04288.x PMC1166865

[B15] Watson JD Sanderson S Ezersky A Savchenko A Edwards A Orengo C Joachimiak A Laskowski RA Thornton JM Towards fully automated structure-based function prediction in structural genomics: a case study J Mol Biol 2007 367 5 1511 1522 10.1016/j.jmb.2007.01.063 17316683 PMC2566530

[B16] Wu S Liang MP Altman RB The SeqFEATURE library of 3D functional site models: comparison to existing methods and applications to protein function annotation Genome Biol 2008 9 R8 10.1186/gb-2008-9-1-r8 18197987 PMC2395245

[B17] Halperin I Glazer DS Wu S Altman RB The FEATURE framework for protein function annotation: modeling new functions, improving performance, and extending to novel applications BMC Genomics 2008 9 Suppl 2 S2 10.1186/1471-2164-9-S2-S2 18831785 PMC2559884

[B18] Binkowski TA Naghibzadeg S Liang J CASTp: computed atlas of surface topography of proteins Nucleic Acids Res 2003 31 3352 3355 10.1093/nar/gkg512 12824325 PMC168919

[B19] Weisel M Proschak E Schneider G PocketPicker: analysis of ligand binding-sites with shape descriptors Chem Cent J 2007 1 7 10.1186/1752-153X-1-7 17880740 PMC1994066

[B20] Nimrod G Schushan M Steinberg DM Ben-Tal N Detection of functionally important regions in "hypothetical proteins" of known structure Structure 2008 16 12 1755 1763 10.1016/j.str.2008.10.017 19081051

[B21] Manikandan K Pal D Ramakumar S Brener NE Iyengar SS Seetharaman G Functionally important segments in proteins dissected using Gene Ontology and geometric clustering of peptide fragments Genome Biol 2008 9 3 R52 10.1186/gb-2008-9-3-r52 18331637 PMC2397504

[B22] Berman HM Westbrook J Feng Z Gilliland G Bhat TN Weissig H Shindyalov IN Bourne PE The Protein Data Bank Nucleic Acids Res 2000 28 235 242 10.1093/nar/28.1.235 10592235 PMC102472

[B23] Ashburner M Ball CA Blake JA Botstein D Butler H Cherry M Davis AP Bolinski K Dwight SS Eppig JT Harris MA Hill DP Issel-Tarver L Kasarskis A Lewis S Matese JC Richardson JE Ringwald M Rubin GM Sherlock G Gene Ontology: tool for the unification of biology Nat Genetics 2000 25 25 29 10.1038/75556 PMC3037419 10802651

[B24] Ebert JC Altman RB Robust recognition of zinc binding sites in proteins Protein Sci 2008 17 54 65 10.1110/ps.073138508 18042678 PMC2144590

[B25] Yoon S Ebert JC Chung EY De Micheli G Altman RB Clustering protein environments for function prediction: finding PROSITE motifs in 3D BMC Bioinformatics 2007 8 Suppl 4 S10 10.1186/1471-2105-8-S4-S10 17570144 PMC1892080

[B26] Giles NM Giles GI Jacob C Multiple roles of cysteine in biocatalysis Biochem Biophys Res Comm 2003 300 1 4 10.1016/S0006-291X(02)02770-5 12480511

[B27] Raychaudhuri S Schutze H Altman RB Using text analysis to identify functionally coherent gene groups Genome Res 2002 12 1582 1590 10.1101/gr.116402 12368251 PMC187532

[B28] Raychaudhuri S Altman RB A literature-based method for assessing the functional coherence of a gene group Bioinformatics 2003 19 3 396 401 10.1093/bioinformatics/btg002 12584126 PMC2669934

[B29] The Uniprot Consortium The Universal Protein Resource (UniProt) Nucleic Acids Res 2008 37 Database D169 74 18836194 10.1093/nar/gkn664 PMC2686606

[B30] PubMed http://www.ncbi.nlm.nih.gov/pubmed

[B31] CYS cluster annotations http://feature.stanford.edu/clustering/cys

[B32] Holm RH Kennepohl P Solomon EI Structural and function aspects of metal sites in biology Chem Rev 1996 96 7 2239 2314 10.1021/cr9500390 11848828

[B33] Messerschmidt A Huber R The blue oxidases, ascorbate oxidase, laccase, and ceruloplasmin. Modelling and structural relationships Eur J Biochem 1990 187 2 341 352 10.1111/j.1432-1033.1990.tb15311.x 2404764

[B34] Auld DS Zinc coordination sphere in biochemical zinc sites Biometals 2001 13 3-4 271 313 10.1023/A:1012976615056 11831461

[B35] Patel K Kumar A Susheel D Analysis of the structural consensus of the zinc coordination centers of metalloprotein structures Biochim Biophys Acta 2007 1774 10 1247 1253 17855175 10.1016/j.bbapap.2007.07.010

[B36] Okada C Maegawa Y Yao M Tanaka I Crystal structure of an RtcB homolog protein (PH1602-extein protein) from Pyrococcus horikoshii reveals a novel fold Proteins 2006 63 4 1119 1122 10.1002/prot.20912 16485279

[B37] Goyal K Mande SC Exploiting 3D structural templates for detection of metal-binding sites in protein structures Proteins 2008 70 4 1206 1218 10.1002/prot.21601 17847089

[B38] Torres-Larios A Sankaranarayanan R Rees B Dock-Bregeon AC Moras D Conformational movements and cooperativity upon amino acid, ATP and tRNA binding in threonyl-tRNA synthetase J Mol Biol 2003 331 201 211 10.1016/S0022-2836(03)00719-8 12875846

[B39] Shaw MA Bond CS Roper JR Gourley DG Ferguson MAJ Hunter WN High-resolution crystal structure of Trypanosoma brucei Udp-Galactose 4'-epimerase: a potential target for structure-based development of novel trypanocides Mol Biochem Parasitol 2004 126 2 173 180 10.1016/S0166-6851(02)00243-8 12615316

[B40] Gu J Milligan J Huang LE Molecular Mechanism of Hypoxia-inducible Factor 1α-p300 Interaction J Biol Chem 2001 276 5 3550 3554 10.1074/jbc.M009522200 11063749

[B41] Kashiwagi T Yokoyama K Ishikawa K Ono K Ejima D Matsui H Suzuki E Crystal Structure of Microbial Transglutaminase from *Streptoverticillium mobaraense* J Biol Chem 2002 277 44252 44260 10.1074/jbc.M203933200 12221081

[B42] Pace HC Brenner C The nitrilase superfamily: classification, structure and function Genome Biol 2001 2 reviews0001.1 0001.9 10.1186/gb-2001-2-1-reviews0001 PMC150437 11380987

[B43] Novo C Farnaud S Tata R Clemente A Brown PR Support for a three-dimensional structure predicting a Cys-Glu-Lys catalytic triad for *Pseudomonas aeruginosa *amidase comes from site-directed mutagenesis and mutations altering substrate specificity Biochem J 2002 365 731 738 11955282 10.1042/BJ20011714 PMC1222709

[B44] Raychaudhuri S Chang JT Imam F Altman RB The computational analysis of scientific literature to define and recognize gene expression clusters Nucleic Acids Res 2003 31 15 4553 4560 10.1093/nar/gkg636 12888516 PMC169898

[B45] Jenkins J Shevchick VE Hugouvieux-Cotte-Pattat N Pickersgill RW The crystal structure of pectate lyase Pel9A from Erwinia chrysanthemi J Biol Chem 2004 279 10 9139 9145 10.1074/jbc.M311390200 14670977

[B46] Smith TF Gaitatzes C Saxena K Neer EJ The WD repeat: a common architecture for diverse functions Trends Biochem Sci 1999 24 5 181 185 10.1016/S0968-0004(99)01384-5 10322433

[B47] Tendulkar AV Joshi AA Sohoni MA Wangikar PP Clustering of protein structural fragments reveals modular building block approach of nature J Mol Biol 2004 338 3 611 629 10.1016/j.jmb.2004.02.047 15081817

[B48] Shindyalov IN Bourne PE An alternative view of protein fold space Proteins 2000 38 3 247 260 10.1002/(SICI)1097-0134(20000215)38:3<247::AID-PROT2>3.0.CO;2-T 10713986

[B49] Tsai CJ Maizel JVJ Nussinov R Anatomy of protein structures: visualizing how a one-dimensional protein chain folds into a three-dimensional shape Proc Natl Acad Sci 2000 97 22 12038 12043 10.1073/pnas.97.22.12038 11050234 PMC17290

[B50] Friedberg I Godzik A Fragnostic: walking through protein structure space Nucleic Acids Res 2005 33 Web Server W249 W251 10.1093/nar/gki363 15980462 PMC1160124

[B51] Neurath H Evolution of proteolytic enzymes Science 1984 224 4647 350 357 10.1126/science.6369538 6369538

[B52] Barrett AJ Proteases Curr Protoc Protein Sci 2001 21 21.1 18429162 10.1002/0471140864.ps2101s21

[B53] Hastie T Tibshirani R Friedman J The Elements of Statistical Learning: Data Mining, Inference, and Prediction 2008 New York: Springer

[B54] Mooney SD Liang MH DeConde R Altman RB Structural characterization of proteins using residue environments Proteins 2005 61 4 741 747 10.1002/prot.20661 16245324 PMC2483305

[B55] Peters B Moad C Youn E Buffington K Heiland R Mooney S Identification of similar regions of protein structures using integrated sequence and structure analysis tools BMC Struct Biol 2006 6 4 16526955 10.1186/1472-6807-6-4 PMC1435900

[B56] de Hoon MJ Imoto S Nolan J Miyano S Open source clustering software Bioinformatics 2004 20 9 1453 1454 10.1093/bioinformatics/bth078 14871861

[B57] lxml - Processing XML and HTML with Python http://codespeak.net/lxml

[B58] Medical Subject Headings (MeSH) Fact Sheet http://www.nlm.nih.gov/pubs/factsheets/mesh.html

[B59] DeLano WL The PyMOL Molecular Graphics System 2008 Palo Alto, CA: DeLano Scientific LLC

