# Supplementary figures and images for: Identification of recurring protein structure microenvironments and discovery of novel functional sites around CYS residues
Source: BMC Struct Biol. 2010 Feb 2;10:4. doi: 10.1186/1472-6807-10-4 (PMC2833161; doi:10.1186/1472-6807-10-4)

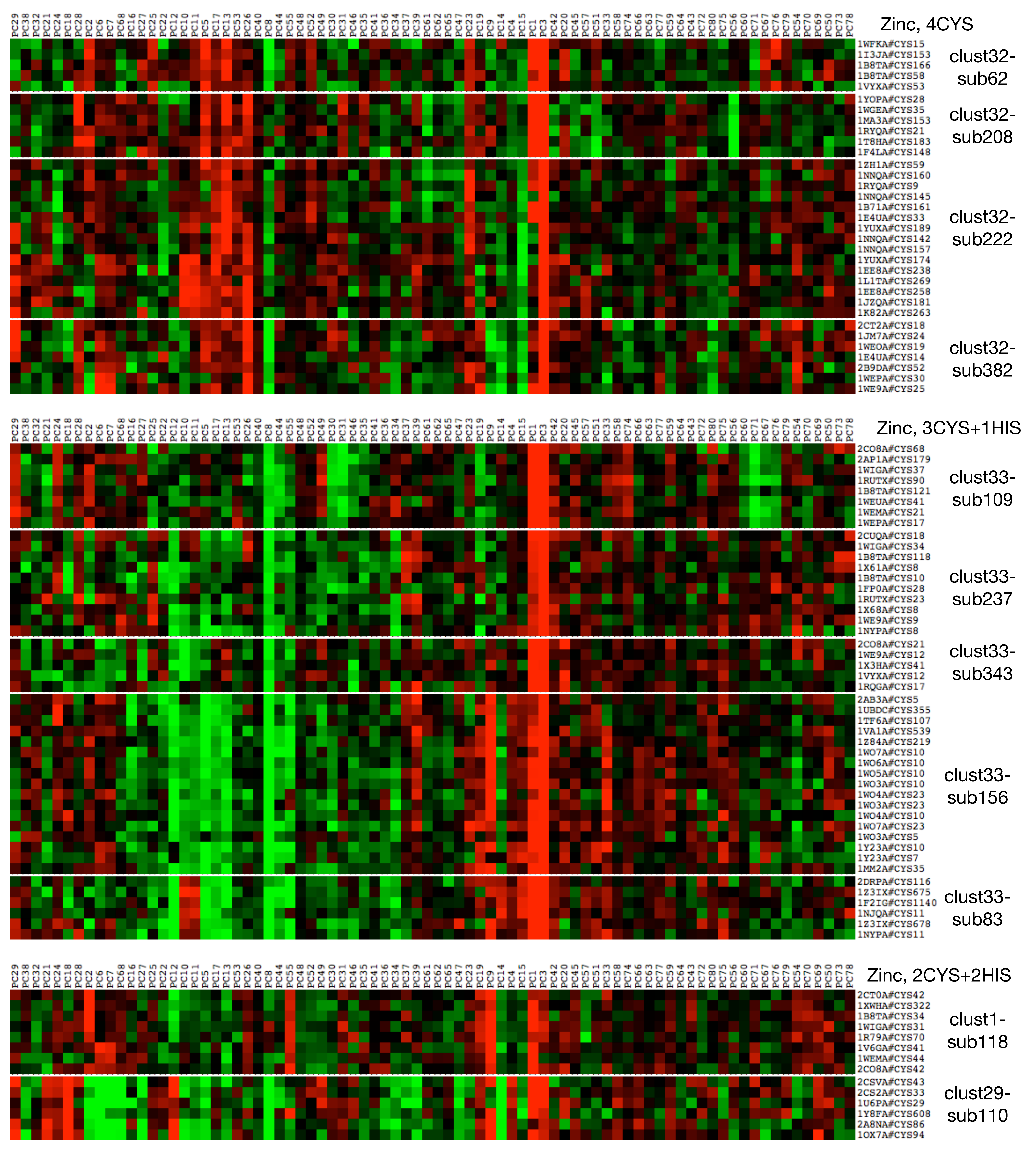

Supplement: Additional file 1 — Figure S1. Feature vectors from similar zinc-binding clusters. We compared feature vectors from clusters corresponding to similar zinc-binding modes - 4CYS (top), 3CYS+1HIS (middle), and 2CYS+2HIS (bottom). The heat maps were generated from a hierarchical clustering of 15 zinc-binding clusters from 4 different coarse clusters. There clearly are major differences between even close clusters like Clust33-Sub60 and Clust33-Sub63. See Additional file 3 - Table S1 for lists of heavily weighted features contributing to the major principal components differing between clusters within similar zinc-binding modes. [file 1472-6807-10-4-S1.PNG]

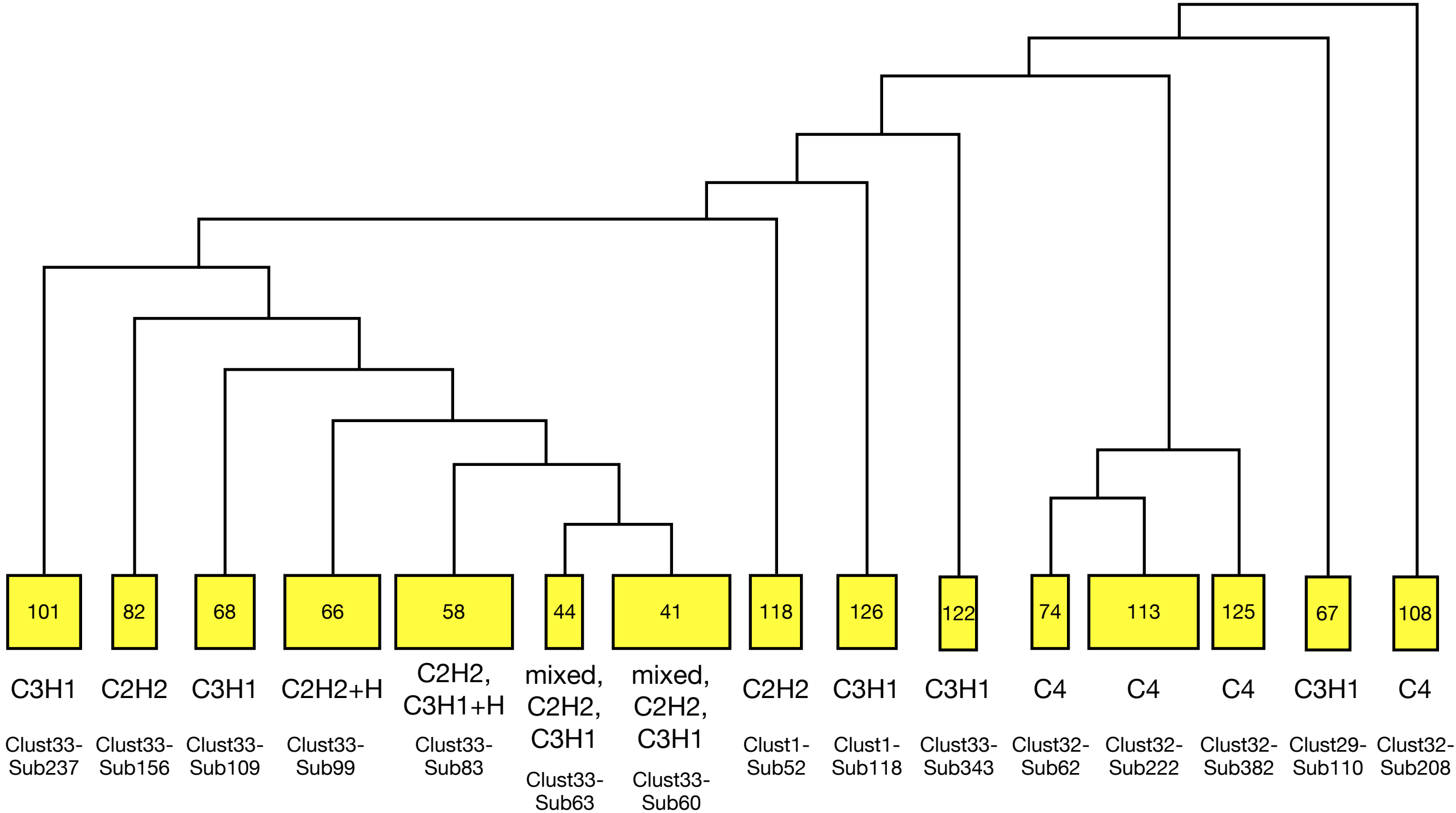

Supplement: Additional file 2 — Figure S2. Hierarchical tree of zinc-binding clusters. We combined the microenvironments from 15 zinc-binding clusters derived from 4 coarse clusters and repeated the cluster selection process. All of the original clusters were found again in the new output with the exception of two microenvironments which became singletons. The tree below (branch lengths not to scale) shows a conceptual ordering between clusters in the new clustering result, with new node labels within the node boxes and original labels below. The zinc binding mode is also indicated below each node (C4 = 4 CYS, C2H2 = 2 CYS + 2 HIS, C3H1 = 3 CYS + 1 HIS, and +H = an additional HIS in the environment not shown as coordinating the ion in the structure). The width of each node box represents the size of that cluster. [file 1472-6807-10-4-S2.PDF]

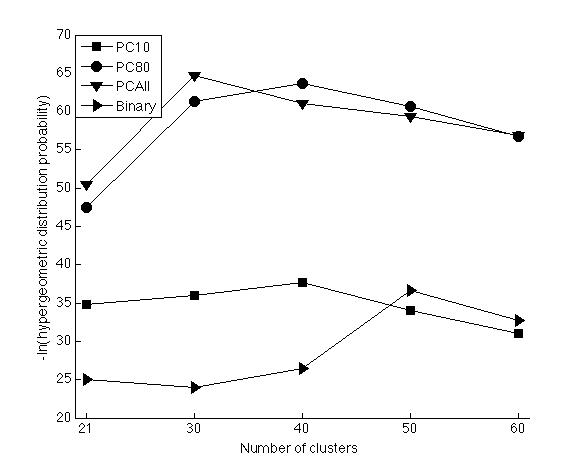

Supplement: Additional file 4 — Figure S3. Hierarchical tree of 15 known functional clusters. We combined the microenvironments from 15 clusters representing training sets for FEATURE models and performed the cluster selection process. The results clearly separate out these 15 clusters (with the two clusters for Alcohol dehydrogenase corresponding to microenvironments centered on two points on the active site tyrosine). The relationships between the 15 clusters in the hierarchical tree may be interesting for further study. (Note: branch lengths are not to scale.) [file 1472-6807-10-4-S4.JPEG]

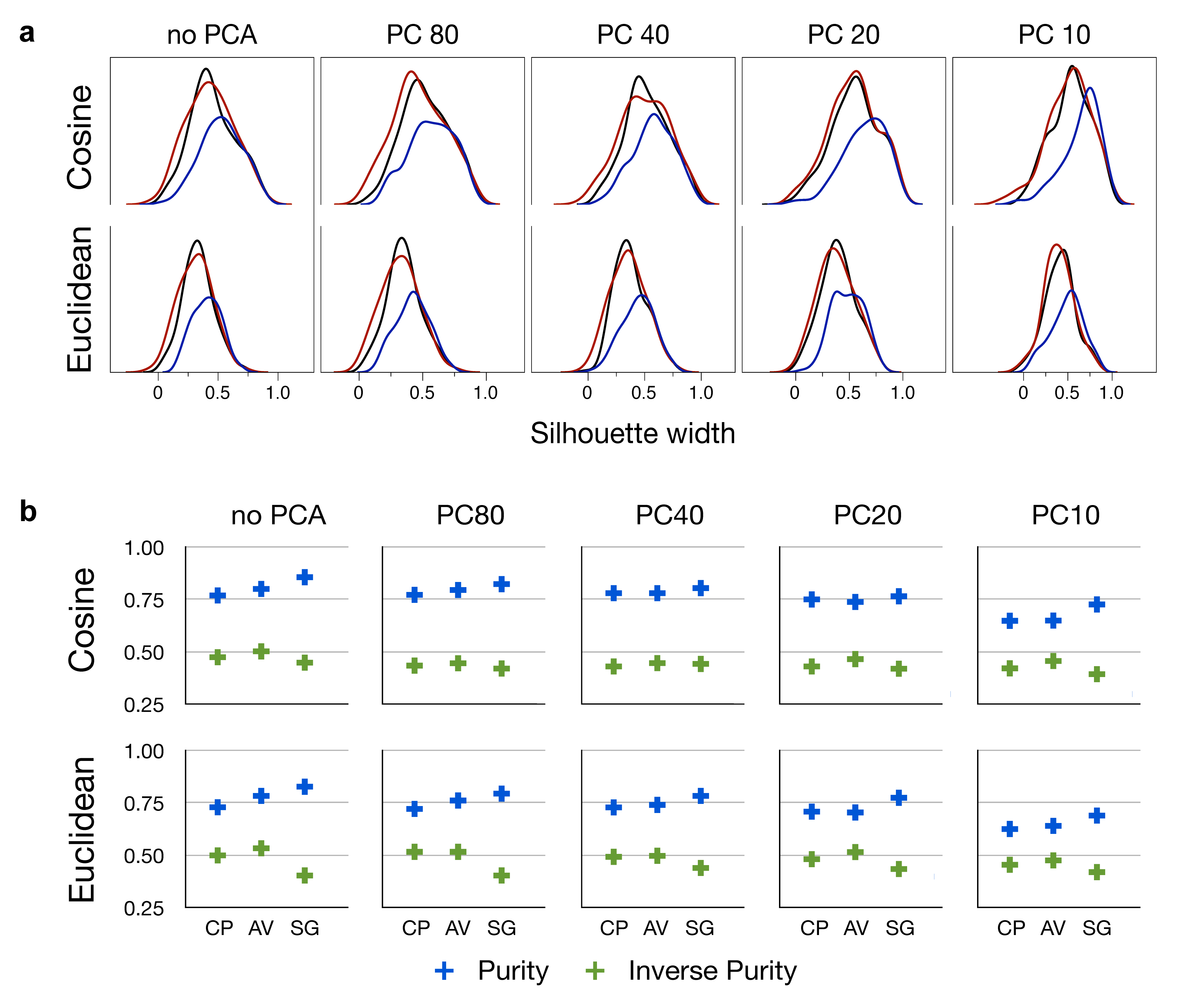

Supplement: Additional file 5 — Figure S4. Parametrization for k-means. To evaluate the optimal number of principal components and value for k, we calculated the average hypergeometric distribution probability of the best represented cluster across each of 21 PROSITE motifs enriched in the CYS data set for each set of parameters. Using 80 principal components with k = 40 resulted in the best performance while still allowing a reduction in the number of dimensions. [file 1472-6807-10-4-S5.PNG]

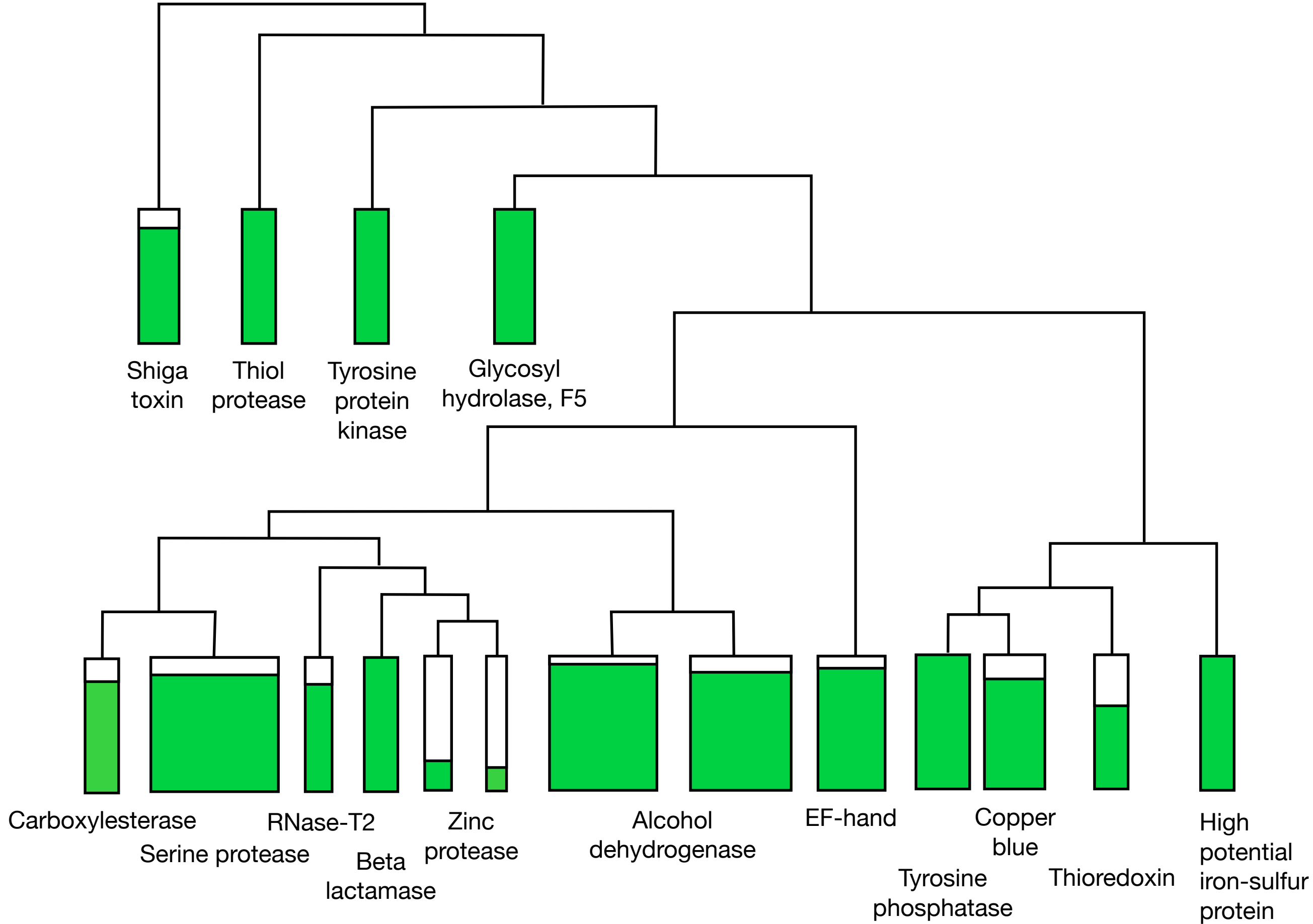

Supplement: Additional file 6 — Figure S5. Single linkage hierarchical clustering with cosine similarity distance metric results in better silhouette widths and precision. We evaluated silhouette widths, purity (precision), and inverse purity (recall) of clusters produced from running the cluster selection process on a test set consisting of about 1400 microenvironments belonging to about 160 PROSITE patterns. We hierarchically clustered vectors corresponding to different numbers of principal components using both cosine similarity and Euclidean distance and using average, complete, or single linkage. (a) We plotted the distribution of silhouette widths resulting from each combination of parameters (blue = single linkage, red = complete linkage, black = average linkage). Single linkage and cosine similarity produce better silhouette distributions for all combinations. (b) We plotted the average inverse purity (recall) and purity (precision) for clusters resulting from each combination of parameters. Single linkage and cosine similarity produce clusters with higher purity. [file 1472-6807-10-4-S6.PDF]
