# Supplementary material for: Identification of recurring protein structure microenvironments and discovery of novel functional sites around CYS residues
Source: BMC Struct Biol. 2010 Feb 2;10:4. doi: 10.1186/1472-6807-10-4 (PMC2833161; doi:10.1186/1472-6807-10-4)
Supplement: Additional file 3 — Table S1. Top contributing features for principal components showing distinct differences between zinc-binding clusters. We expect to see differences between clusters binding zinc with different residues, but many distinct clusters actually bind zinc in the same way. This table shows the features that contribute the most to the differences between distinct clusters binding zinc in the same way. Each column contains six principal components that showed the most variation between different clusters with the same type of zinc binding. For clusters binding zinc with 4 CYS, principal components 2, 10, 56, 54, 51, and 39 differed the most between them; for clusters binding zinc with 3 CYS and 1 HIS, components 10, 9, 71, 25, 49, and 2 differed the most; and for clusters binding zinc with 2 CYS and 2 HIS, components 2, 12, 54, 55, 39, and 7 differed the most. The top five most heavily weighted features for each principal component are shown with the feature name followed by its weight in that component. These observations demonstrate that even sub-types of zinc binding can be delineated further on the basis of less obvious features. [file 1472-6807-10-4-S3.PDF]

4CYS

PC2	
Aromatic carbon-3	-0.17294
Carboxyl oxygen-1	0.17098
Carboxyl oxygen-0	0.16968
Residue name is GLY-4	-0.16518
Residue name is GLY-3	-0.16192

PC10	
Residue name is other-3	0.24717
Residue name is other-4	0.24299
Residue name is other-5	0.24261
Residue name is other-2	0.23245
Residue name is LYS-4	0.22677

PC56	
Residue name is ARG-2	-0.44874
Residue name is ARG-3	0.39914
Residue name is ARG-4	-0.29489
Residue name is TYR-2	-0.21303
Hydrophobicity-5	-0.18064

PC54	
Residue name is ARG-2	-0.25273
Amide nitrogen-5	-0.24303
Charge on histidines-2	0.22694
Amide nitrogen-3	0.22114
Aromatic carbon-2	0.21132

PC51	
Van der Waals volume-1	-0.32825
Residue name is ARG-2	-0.28619
Aromatic carbon-1	-0.20179
Residue name is ARG-5	-0.20162
Residue name is GLN-2	-0.17471

PC39	
Residue name is HIS-5	0.21069
Residue name is HIS-4	-0.18425
Residue name is ASP-5	0.18173
Aliphatic carbon-0	0.17623
Aliphatic carbon-1	-0.17552

3CYS 1HIS

PC10	
Residue name is other-3	0.24717
Residue name is other-4	0.24299
Residue name is other-5	0.24261
Residue name is other-2	0.23245
Residue name is LYS-4	0.22677

PC9	
Residue name is CYS-2	-0.21654
Residue name is ILE-5	-0.20947
Residue name is GLU-2	0.19422
Charge-4	0.18859
Charge-5	0.17616

PC71	
Residue name is PHE-5	0.34488
Residue name is PHE-2	-0.32597
Residue name is THR-2	0.28784
Hydroxyl oxygen-5	-0.23183
Residue name is THR-5	-0.22559

PC25	
Residue name is VAL-3	0.25232
Residue name is VAL-4	0.23929
Residue name is VAL-2	0.20508
Residue name is VAL-5	0.19319
Solvent accessibility-4	-0.18985

PC49	
Aromatic carbon-2	0.25811
Aliphatic carbon next to a polar atom-3	-0.25599
Residue name is CYS-4	-0.24112
Aliphatic carbon next to a polar atom-2	0.22509
Residue name is CYS-3	0.21467

PC2	
Aromatic carbon-3	-0.17294
Carboxyl oxygen-1	0.17098
Carboxyl oxygen-0	0.16968
Residue name is GLY-4	-0.16518
Residue name is GLY-3	-0.16192

2CYS 2HIS

PC2	
Aromatic carbon-3	-0.17294
Carboxyl oxygen-1	0.17098
Carboxyl oxygen-0	0.16968
Residue name is GLY-4	-0.16518
Residue name is GLY-3	-0.16192

PC12	
Carboxyl oxygen-3	-0.20071
Aromatic nitrogen-5	0.18918
Residue name is LEU-3	-0.18608
Residue name is GLY-5	0.17524
Carboxyl carbon-5	0.17483

PC54	
Residue name is ARG-2	-0.25273
Amide nitrogen-5	-0.24303
Charge on histidines-2	0.22694
Amide nitrogen-3	0.22114
Aromatic carbon-2	0.21132

PC55	
Amide carbon-3	0.40566
Amide carbon-2	-0.39721
Charge-2	-0.29998
Amide carbon-5	-0.21518
Residue name is GLN-3	0.21282

PC39	
Residue name is HIS-5	0.21069
Residue name is HIS-4	-0.18425
Residue name is ASP-5	0.18173
Aliphatic carbon-0	0.17623
Aliphatic carbon-1	-0.17552

PC7	
Residue name is LEU-1	0.28759
Positively charged nitrogen-1	0.27183
Residue name is ILE-1	-0.23594
Partial charge-1	0.2304
Amide nitrogen-1	0.21467
